# Insomnia Comorbid With Depression: A Bibliometric and Visualized Analysis of Research Trends and Hotspots From 2000 to 2024

**DOI:** 10.1155/bn/7106629

**Published:** 2025-03-08

**Authors:** Junting Chen, Cai Li, Ling Chen, Ziyi Zhao, Yongsu Zheng, Xiaoyan Yang, Hao Huang, Renli Deng

**Affiliations:** ^1^Department of Neurology, Affiliated Hospital of Zunyi Medical University, Zunyi, Guizhou, China; ^2^Department of Nursing, Zunyi Medical University, Zunyi, Guizhou, China

**Keywords:** bibliometric analysis, comorbidity, depression, insomnia, visualization

## Abstract

**Background:** Sleep disorders, such as insomnia, are pervasive and frequently comorbid with depression, significantly affecting the quality of life of patients. Insomnia is characterized by difficulty initiating or maintaining sleep, which leads to impairment. Depression is characterized by persistent sadness and loss of interest, and it often features symptoms of insomnia. Understanding their interaction will be done for treatment strategies concerning both disorders. Despite the existence of extensive studies on insomnia and depression, there is a significant gap in bibliometric analysis specifically addressing the comorbidity of these two conditions.

**Objectives:** This study is aimed at conducting a bibliometric analysis of research in insomnia comorbid with depression (ICD) to identify research trends, collaboration patterns, influential works, and hotspots.

**Methods:** The study involved performance analysis to evaluate research productivity and trends, science mapping to visualize the intellectual structure and thematic evolution of the field, and network analysis to examine research collaboration and knowledge structure. Tools such as VOSviewer, CiteSpace, and GraphPad Prism were utilized for data analysis.

**Results:** A total of 1624 publications on the comorbidity of insomnia and depression from 2000 to 2024 were included, encompassing both articles and reviews. Publication volume showed a steady growth from 2000 to 2008, followed by a significant increase from 2019 onward. The United States was the most productive country, followed by China. Key authors such as Allison G. Harvey, Charles M. Morin, and Daniel J. Buysse have made substantial contributions to the field. Major influential journals included *Sleep Medicine*, *Journal of Affective Disorders*, and *Frontiers in Psychiatry*. Research trends identified included the exploration of neurobiological mechanisms, cognitive behavioral therapy for insomnia (CBT-i), and personalized treatment approaches.

**Conclusion:** This bibliometric analysis provides valuable insights into the evolving landscape of research on ICD. Future research should focus on personalized, multimodal interventions, expanding the application of CBT-i, exploring neurobiological mechanisms, and improving patients' quality of life through integrated treatment strategies.

## 1. Introduction

Sleep disorders represent an extensive range of conditions that alter standard sleep patterns and thus negatively affect the health and diurnal functioning of a person. Accordingly, the ICSD-3 main groups for sleep disorders include insomnia, sleep-related breathing disorders, central disorders of hypersomnolence, circadian rhythm sleep-wake disorders, parasomnias, and sleep-related movement disorder [1]. Insomnia is the most common type of sleep disorder, followed and diffused by difficulties initiating/maintaining sleep or waking up too early in the morning. It is diagnosed based on its persistence and associated daytime impairment, which includes fatigue, mood disturbances, and cognitive difficulties. Approximately 10% of adults have insomnia disorder, while another 20% show occasional symptoms. At-risk groups include women, older adults, and those with socioeconomic difficulties. Insomnia tends to become chronic, with a 40% persistence rate over 5 years, and also leads to increased risks for mental health problems, physical health issues, occupational health issues, and a significant economic burden from direct treatment costs and indirect lost work productivity and reduced employment [2].

Depression is a common mental disorder characterized by persistent sadness, loss of interest, and difficulty concentrating [3]. Over time, the symptoms may include anhedonia, psychomotor dysfunction, sleep and appetite disturbances, concentration problems, and suicidal thoughts. According to the WHO estimates, depression affects 3.8% of the global population, including 5.0% of adults and 5.7% of adults over 60 years old. This accounts for approximately 280 million people worldwide. Depression is a leading cause of disability and a significant contributor to the global disease burden. The prevalence of depression is higher in women than in men [4].

Study have shown that insomnia and depression are highly intertwined. Most of the time, insomnia is a premier symptom of depression. About 40%–75% of subjects with significant depression meet the diagnostic criteria for clinical insomnia. Furthermore, in addition to being a symptom, insomnia is also an independent risk factor for the development of depression. This comorbid relationship indicates that insomnia can both precede and exacerbate depressive episodes [5]. Several pathophysiological mechanisms account for the comorbid relationship between depression and insomnia. Initially, there is neurotransmitter imbalance, which is crucial in both conditions. Patients with depression typically evidence anomalies in serotonin, norepinephrine, and also dopamine levels, which represent otherwise known biochemical markers of change in people with insomnia [6]. Secondly, depression is often associated with hyperactivation of the hypothalamic–pituitary–adrenal (HPA) axis, leading to elevated cortisol levels. This stress hormone can also disrupt sleep by causing difficulties in sleep onset and maintenance [7, 8]. In addition, chronic inflammation can be considered one common pathologic feature shared between depression and insomnia. High inflammatory markers, such as C-reactive protein and cytokines, exist in many patients who are depressed and may influence normal sleep by disturbing the central nervous system [9, 10]. Despite extensive studies on both insomnia and depression individually, there is a significant gap in bibliometric analyses specifically addressing the comorbidity of these two conditions. Predecessors have mainly been linked with the clinical or epidemiological aspects of the problem, but there is no complete bibliometric analysis that could provide scope for studying research trends, collaboration patterns, and influential works related to this area.

Bibliometric analysis is a quantitative method for assessing academic literature. By using software like VOSviewer and CiteSpace, the results are visualized, providing insights into scientific trends, influential publications, and research collaboration patterns [11, 12]. Bibliometric analysis consists of three main components: performance analysis, science mapping, and network analysis. Performance analysis evaluates research productivity by analyzing publication output; citation impact; and contributions of authors, organizations, countries, and journals. Science mapping visualizes the intellectual structure of a research field, identifying key themes, research hotspots, and evolving trends using techniques such as keyword co-occurrence and citation network analysis. Network analysis examines relationships among research entities, such as author collaborations, organizational partnerships, and journal cocitation networks, helping to reveal influential researchers, global collaboration patterns, and research clusters [13]. The method has applications in biomedicine, yielding solid diagnostic and treatment bases for many diseases [14, 15]. Our study offers valuable insights into the scientific landscape of insomnia and depression comorbidity studies, highlighting emerging areas and potential gaps. Therefore, an in-depth bibliometric analysis is essential to delineate the scope of existing research, identify influential studies and hotspots, and suggest future research trend and priorities, ultimately contributing to more integrated and effective clinical interventions.

## 2. Materials and Methods

### 2.1. Data Sources and Publications Selection

**Figure 1:**
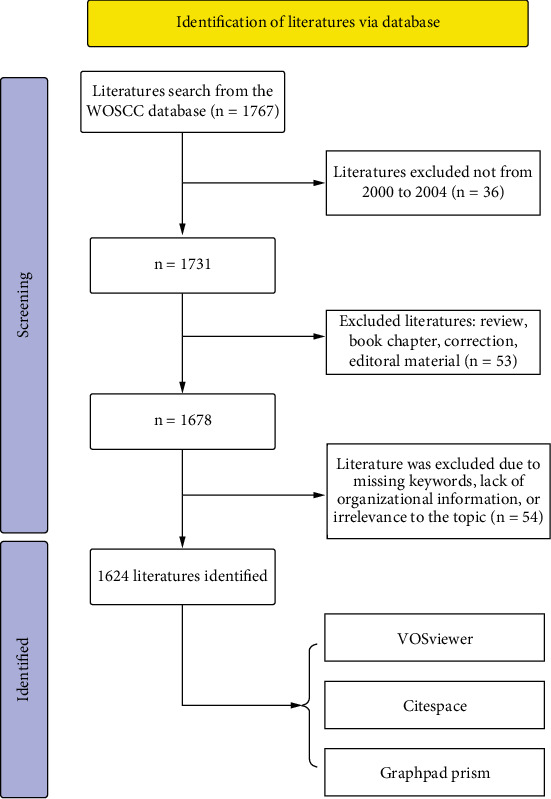
The selection of publications flow chart.

Web of Science Core Collection (WoSCC) is chosen for bibliometric analysis due to its comprehensive coverage of high-quality, peer-reviewed journals across various disciplines [16]. It offers detailed citation data essential for identifying influential research, authors, and trends. With advanced search capabilities and well-curated, standardized data, WoSCC ensures accuracy and reliability in bibliometric studies [17]. We used ((((((((TS = (Depression)) OR TS = (“Depressive Symptoms”)) OR TS = (“Depressive Symptom”)) OR TS = (“Symptom, Depressive”)) OR TS = (“Emotional Depression”)) OR TS = (“Depression, Emotional”)) OR TS = (“Depressive Disorder”)) OR TS = (“Bipolar Disorder”)) OR TS = (“Dysthymic Disorder”) AND ((((((((((TS = (Insomnia∗)) OR TS = (“Insomnia Disorder”)) OR TS = (“Chronic Insomnia”)) OR TS = (“Nonorganic Insomnia”)) OR TS = (“Insomnia, Nonorganic”)) OR TS = (“Primary Insomnia”)) OR TS = (“Psychophysiological Insomnia”)) OR TS = (“Insomnia, Psychophysiological”)) OR TS = (“Secondary Insomnia”)) OR TS = (“Transient Insomnia”)) OR TS = (“Insomnia, Transient”) AND (TS = (“comorbid”) OR TS = (“comorbidity”) OR TS = (“co-occurrence”)). The literature screening for our study was conducted based on the following inclusion criteria: (1) full-text publications related to ICD; (2) articles were written in English; and (3) articles published between January 1, 2000, and December 31, 2024. The exclusion criteria were as follows: (1) studies unrelated to the topic of ICD and (2) articles categorized as conference abstracts, news items, bulletins, or other similar types. The extracted literatures were provided in plain text format. Initial screening of titles and abstracts was conducted by three independent researchers, and the results were reviewed for relevance by two other researchers. Literature that lacked keywords, authors, organizations, and abstracts or was deemed irrelevant to the study topic was excluded. Inconsistencies were resolved by majority vote. After rigorous screening, a total of 1624 valid articles were identified for final analysis.  Table 1 and Figure 1 detail the search strategy and processing.

### 2.2. Data Analysis

We conducted a comprehensive bibliometric analysis based on Donthu et al., incorporating performance analysis, science mapping, and network analysis [13]. The software tools used included GraphPad Prism (version 8.0.2), CiteSpace (version 6.2.4R), and VOSviewer (version 1.6.18).

### 2.3. Performance Analysis

Performance analysis was conducted to evaluate publication trends, key contributing organizations, and journal impact. GraphPad Prism was used to plot annual publication trends from 2000 to 2024 and maps the total number of publications per country in the field. VOSviewer was employed to analyze organizational contributions by constructing a co-occurrence network based on publication volume, providing insights into research productivity and collaboration patterns. To identify the most influential references, a cocited reference analysis was conducted using CiteSpace. CiteSpace was used to detect the most frequently cocited references and to visualize their relationships within a cocitation network. Subsequently, we performed a cocited reference cluster analysis and a timeline analysis to track the evolution of the research themes. Additionally, we analyzed the impact of journals in this field. VOSviewer's density map was utilized to visualize the publication volume of different journals, while CiteSpace was used to generate a journal cocitation network map, identifying the most influential sources in the domain.

### 2.4. Science Mapping

Science mapping was performed to visualize the intellectual landscape and thematic evolution of research on ICD, describe the content of each thematic cluster, and predict future research trends in the field based on keyword analysis. VOSviewer was used to conduct keyword co-occurrence analysis, revealing dominant research themes and their interconnections. In order to track the evolution of research hotspots over time, we used CiteSpace's timezone view (also known as “peak map”) to show the process of their emergence and development, and based on keyword clustering analysis, we identified the main research themes and their evolutionary trends. Also, based on the journals, we developed a dual-map overlay for identifying major research themes and their evolutionary trends. Meanwhile, to detect emerging research frontiers, burst analysis of both cocited references and keywords was conducted using CiteSpace, enabling us to identify recent high-impact publications and emerging research topics.

### 2.5. Network Analysis

Network analysis was conducted to examine collaborative structures among authors, organizations, and journals. CiteSpace was employed to construct an organizational collaboration network, mapping research collaborations among major contributing organizations. This network illustrated global academic partnerships and identified leading organizations in the field. Instead of bibliographic coupling (which focuses on shared references), our study analyzed cocited references using CiteSpace, constructing a cocitation network that grouped references based on their cocitation patterns. This approach highlighted the key knowledge structure of the field.

## 3. Results

### 3.1. Publication Outputs

Our study found that from January 1, 2000, to December 31, 2024, a total of 1624 publications on ICD were indexed in the WoSCC database. These publications involved 81 countries/regions, 2405 organizations, and 7816 authors.

**Figure 2:**
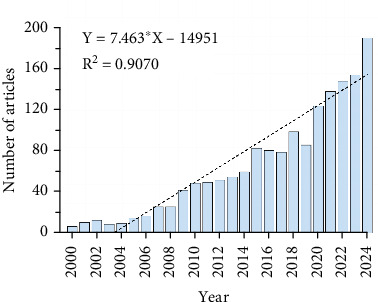
Annual volume of publications.

Since 2000, the annual number of publications has shown a gradual increase (Figure 2). We divided this trend into three phases: From 2000 to 2008, the growth was slow, with an average of about 30 publications per year, indicating limited attention to this field by researchers; from 2009 to 2019, the number of publications gradually increased; and after 2019, the growth accelerated further, reaching its peak in 2024.

### 3.2. Analysis of Top Productive Countries/Regions and Organizations

**Figure 3:**
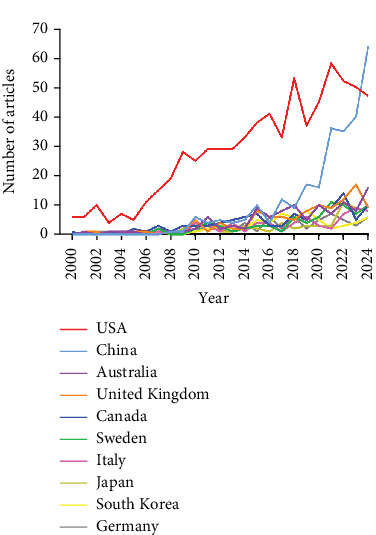
Line graph of national publications.

**Figure 4:**
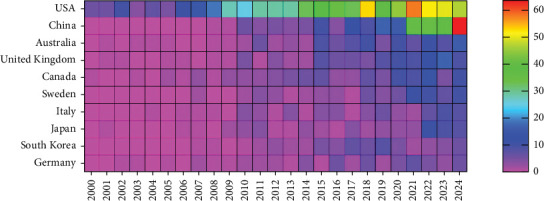
Heat map of national publications.

**Figure 5:**
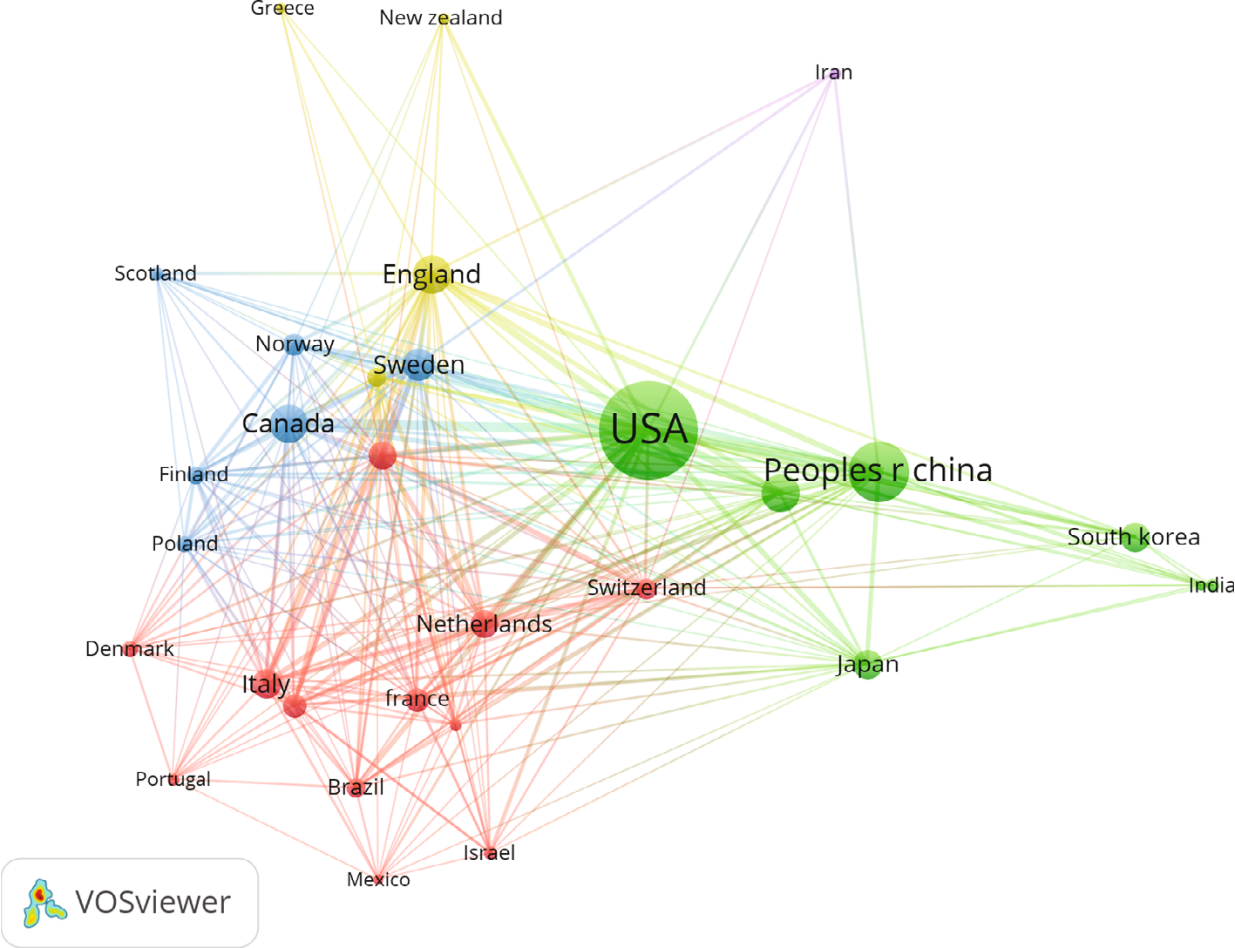
Networks of country cooperation.

Research on the application of studies related to ICD has been conducted in 81 countries/regions. Figures 3 and 4 illustrate the annual publication trends of the top 10 countries over the past decade. The top five countries in this field are the United States, China, Australia, the United Kingdom, and Canada. The United States stands out significantly, contributing 43.66% of the total publications, far exceeding the output of other countries. Among the top 10 countries/regions in terms of publication volume, the United States has the highest citation count, with 32,894 citations (Table 2). Its citations per publication ratio (46.39) rank fifth among all countries, indicating a generally high quality of published papers. China ranks second in publication volume (269 papers) but fourth in total citations (5224), with a relatively low citation per publication ratio (19.4). The collaboration network, as shown in Figure 5, highlights close collaboration between the two leading countries, the United States and China. The United States also maintains strong collaborations with Germany, Sweden, and Italy, while China shows closer ties with Japan, South Korea, and Australia. The United States not only has the highest publication volume but also the highest citation frequency, demonstrating its leadership in this field.

**Figure 6:**
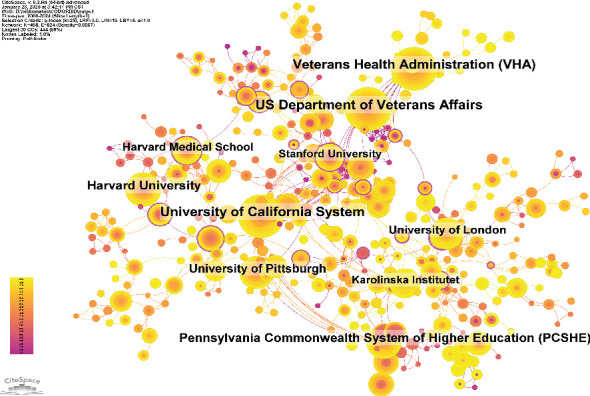
Networks of organizational cooperation.

A total of 2405 organizations has systematically published articles on ICD. Among the top 10 organizations in terms of publication volume, there are nine organizations from the United States, and only one organization is from the United Kingdom (Table 3 and Figure 6). The University of California System published the most articles (93 papers, 4045 citations, with an average of 43.49 citations per paper). The US Department of Veterans Affairs ranked second (79 papers, 8026 citations, with an average of 101.59 citations per paper), followed by the Veterans Health Administration (56 papers, 3623 citations, with an average of 64.70 citations per paper). Harvard University ranked fourth, with 53 papers and the highest average citation count (9160 citations, 172.83 citations per paper). Further analysis revealed that organizations, both domestic and international, tend to collaborate more frequently with organizations within their own country. To advance the field and overcome academic barriers, we advocate for stronger collaboration between domestic and international organizations.

### 3.3. Analysis of Journal

**Figure 7:**
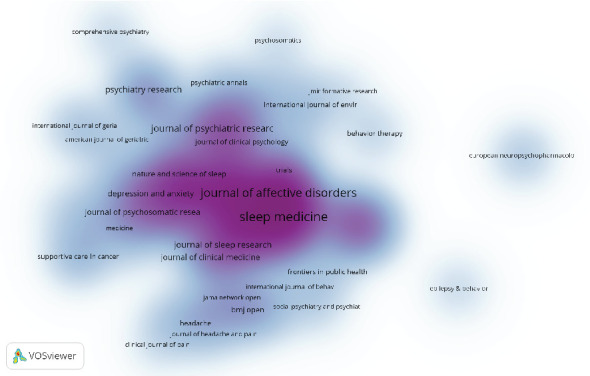
Density map of journal publication.

**Figure 8:**
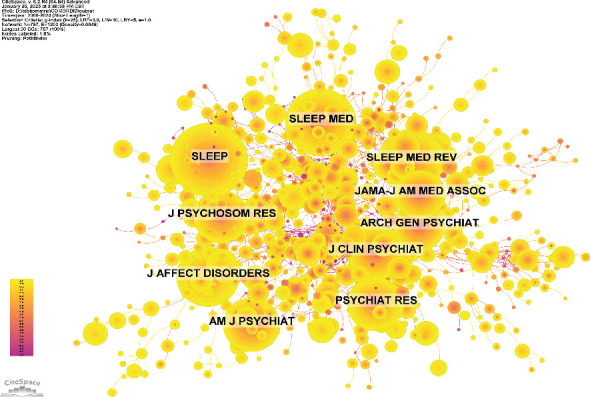
Cocitation network map of journal.

The top 10 journals with the highest publication volume (Table 4) and citation counts (Table 5) are listed. *Sleep Medicine* leads the field with 74 articles (4.56%), followed by the *Journal of Affective Disorders* with 56 articles (3.45%), *Sleep* with 52 articles (3.20%), and *Frontiers in Psychiatry* with 36 articles (2.22%). Among these prolific journals, *Sleep Medicine Reviews* has the highest impact factor (IF) of 11.2. Notably, 90% of these journals are classified within the Q1 or Q2 quartiles, indicating their high standing in the field. The number of journal publications was also presented visually, as shown in Figure 7. The impact of a journal is determined by the frequency of its cocitations, indicating its significant influence on the scientific community. According to Figure 8 and Table 5, the most cocited journal is *Sleep* (1149 cocitations), followed by *Sleep Med* (911 cocitations) and *Sleep Med Rev* (856 cocitations). Among the top 10 most cocited journals, *JAMA-J Am Med Assoc* received 582 cocitations and has the highest IF of 63.5. Notably, 90% of the cocited journals are classified in the Q1/Q2 quartiles.

**Figure 9:**
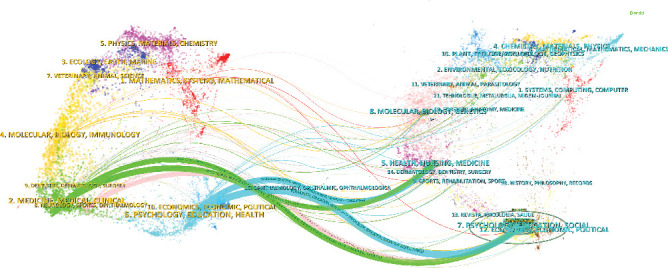
Dual map of journal.

The thematic distribution of academic publications is visualized using a dual-map overlay (Figure 9). The colored pathways represent citation links, where citing journals are displayed on the left and cited journals on the right. Based on the results, we identified some primary citation pathways: (1) Research published in journals within the health/nursing/medicine domain is primarily cited by studies published in psychology/education/health and medicine/medical/clinical journals; (2) research from psychology/education/social journals is predominantly cited by psychology/education/health, neurology/sports/ophthalmology, and medicine/medical/clinical journals.

### 3.4. Analysis of Authors and Cocited Authors

**Figure 10:**
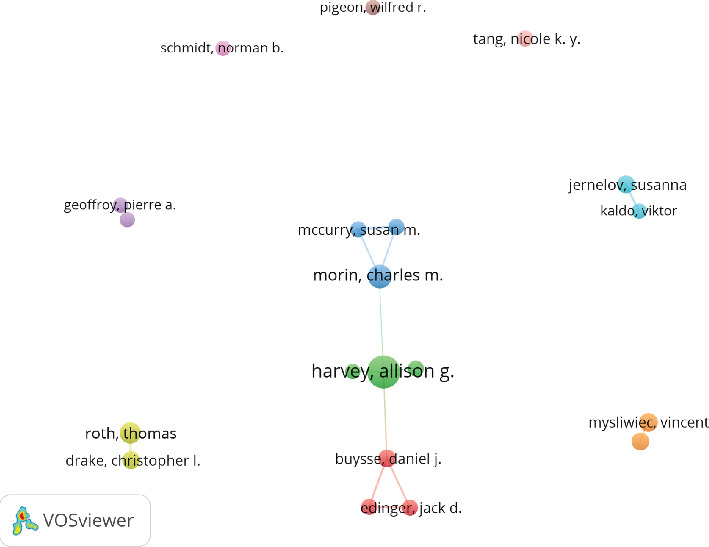
Cooperation network of author.

**Figure 11:**
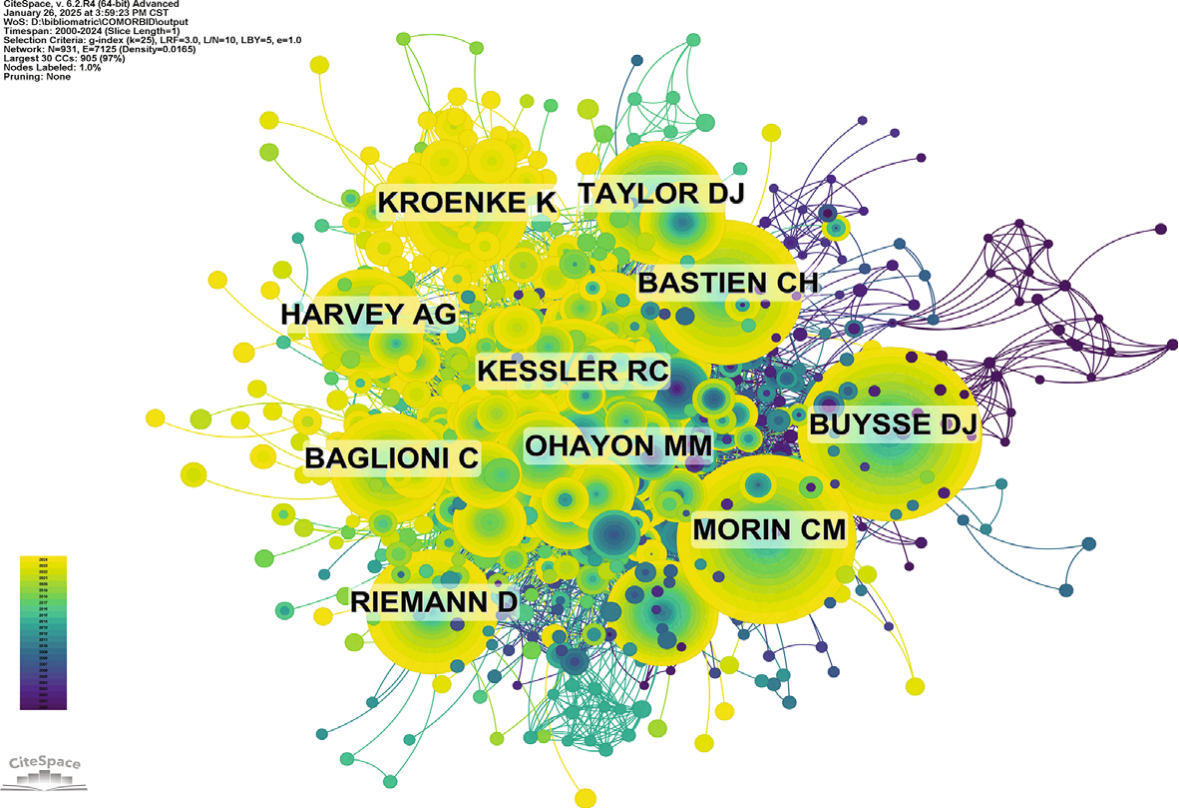
Cocitation network of author.

Among all authors who have published literature related to ICD, Table 5 lists the top 10 most prolific authors. These 10 authors collectively contributed 121 papers, accounting for 7.45% of all publications in this field. The most published author is Allison G. Harvey, with 24 papers, followed by Charles M. Morin (15 papers), Thomas Roth (13 papers), and Vincent Mysliwiec (11 papers). The collaboration network among authors is visualized using CiteSpace (Figure 10), illustrating their interconnections.  Figure 11 and Table 6 present the top 10 most cocited and most cited authors, respectively. A total of 64 authors have been cited more than 50 times, indicating their high reputation and significant influence in the field. The largest nodes in the cocitation network correspond to the most frequently cocited authors, including Daniel J. Buysse (499 citations), Charles M. Morin (485 citations), and M. M. Ohayon, (416 citations). Further analysis reveals that while Daniel J. Buysse ranks fourth in publication volume, he holds the top position in total citations, underscoring his leadership and substantial impact in this research domain.

### 3.5. Analysis of Cocited References

**Figure 12:**
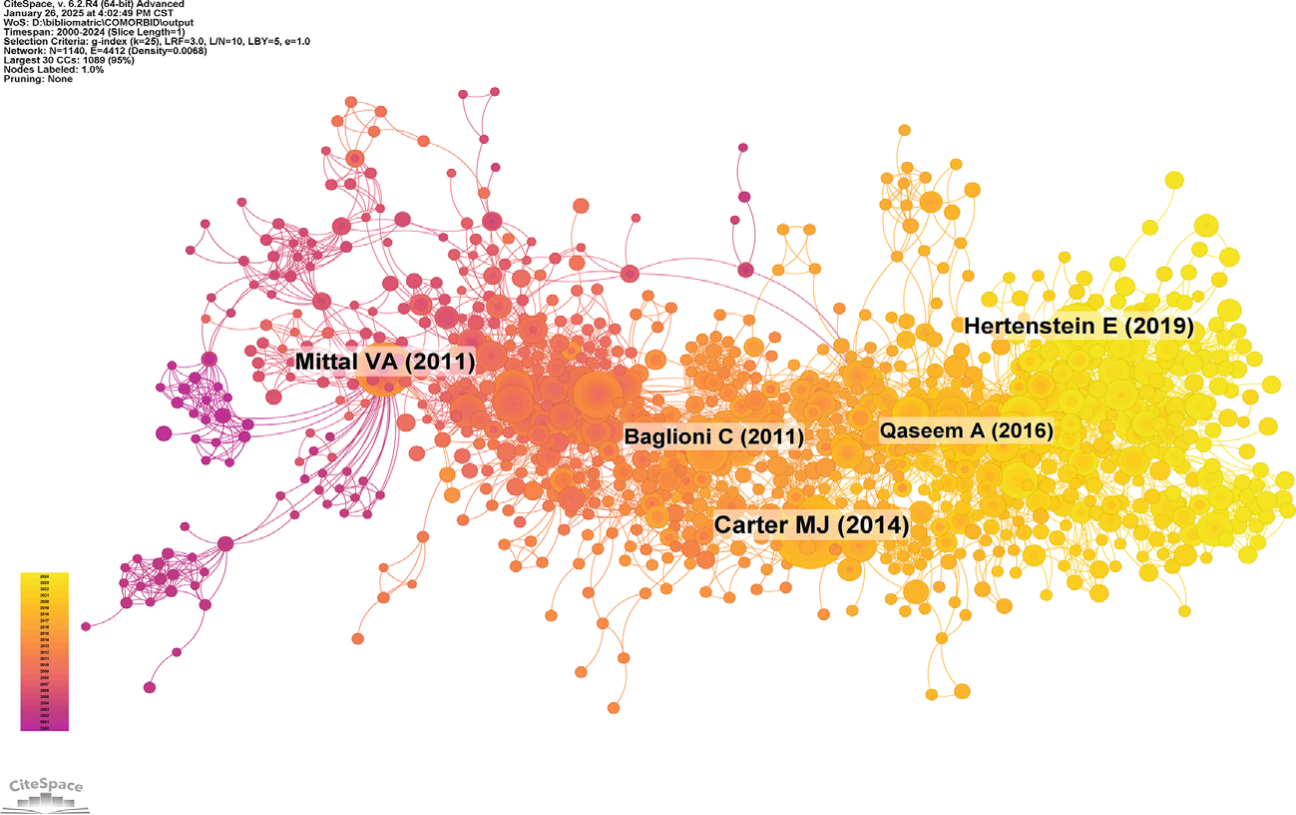
Cocited network of reference.

With a 1-year time slice from 2000 to 2024, the cocitation reference network consists of 1140 nodes and 4412 links (Figure 12). According to the top 10 most cocited articles (Table 7), the study titled “Insomnia as a Predictor of Mental Disorders: A Systematic Review and Meta-Analysis” published in *Sleep Medicine Reviews* is the most frequently cocited paper. It systematically examined whether insomnia serves as a predictor not only for depression but also for other mental disorders. While previous research has established a strong association between insomnia and depression, this study expanded on that understanding by including data from 13 longitudinal studies with a total sample of 181,798 participants. The findings demonstrated that insomnia is not only a significant predictor of depression (OR = 2.83, CI 1.55–5.17) but substantially increases also the risk of developing anxiety disorders (OR = 3.23, CI 1.52–6.85), alcohol abuse (OR =1.35, CI 1.08–1.67), and psychosis (OR = 1.28, CI 1.03–1.59). These results suggest that insomnia may serve as a common risk factor for multiple psychiatric disorders rather than merely a comorbid condition of depression. Moreover, the study employed strict inclusion criteria, selecting only studies that assessed baseline insomnia (including both nighttime and daytime symptoms) with a follow-up period of at least 12 months, thereby enhancing the reliability of the results. Although the study provides strong evidence of an association between insomnia and the onset of mental disorders, the causal relationship remains to be further elucidated. Future research should incorporate more prospective studies with rigorous diagnostic criteria, extended follow-up durations, and a broader range of psychiatric disorders to validate the predictive value of insomnia in mental health outcomes [18].

The second-ranked article is “Insomnia as a Predictor of Depression: A Meta-Analytic Evaluation of Longitudinal Epidemiological Studies,” published in the *Journal of Affective Disorders* and authored by Baglioni et al. [19] who conducted a meta-analytic evaluation of 21 longitudinal epidemiological studies to systematically examine whether insomnia serves as an independent predictor of depression. Previous research has established a strong link between insomnia and depression, with insomnia often preceding the onset of depressive episodes and, in some cases, persisting even after effective treatment. However, this study expands upon existing knowledge by quantitatively assessing the predictive role of insomnia in depression, rather than simply viewing it as a symptom. Through a comprehensive literature search spanning from 1980 to 2010 across multiple databases (PubMed, Medline, PsycInfo, and PsycArticles), the authors identified studies that investigated both insomnia symptoms and depressive psychopathology over time. Using both fixed-effects and random-effects meta-analytic models, they found that individuals with insomnia had a significantly elevated risk of developing depression, with the random-effects model yielding an overall odds ratio of 2.60 (CI: 1.98–3.42). After adjusting for outliers, the studies were no longer heterogeneous, and the fixed-effects model provided a more conservative estimate of an odds ratio of 2.10 (CI: 1.86–2.38), indicating that individuals with baseline insomnia had a twofold increased risk of developing depression compared to those without sleep disturbances. These findings contribute significantly to the field of insomnia and comorbid depression by reinforcing the notion that insomnia is not merely a consequence or symptom of depression but rather an independent risk factor for its onset. This perspective supports the growing shift away from conceptualizing insomnia as a secondary condition and instead recognizing it as a clinically relevant predictor of psychiatric morbidity [19].

**Figure 13:**
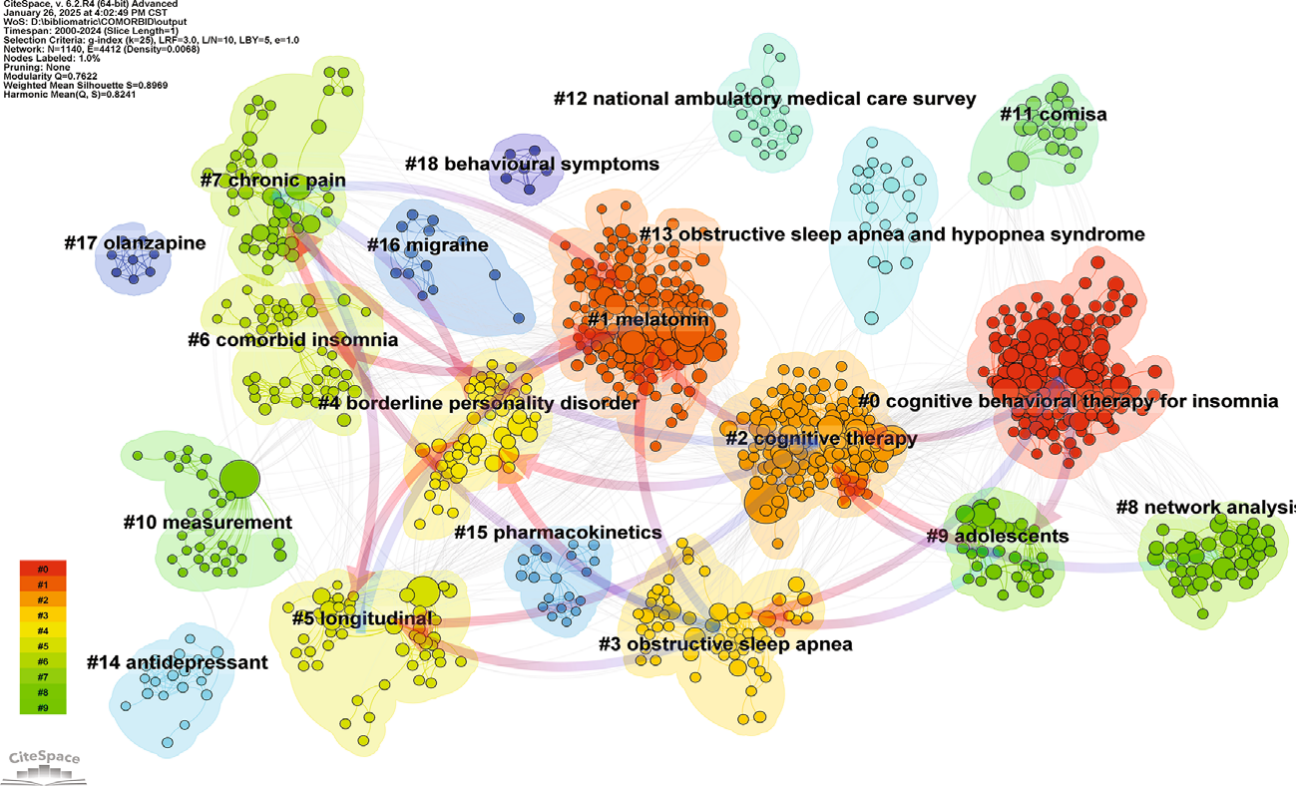
Clustering of cocited reference.

**Figure 14:**
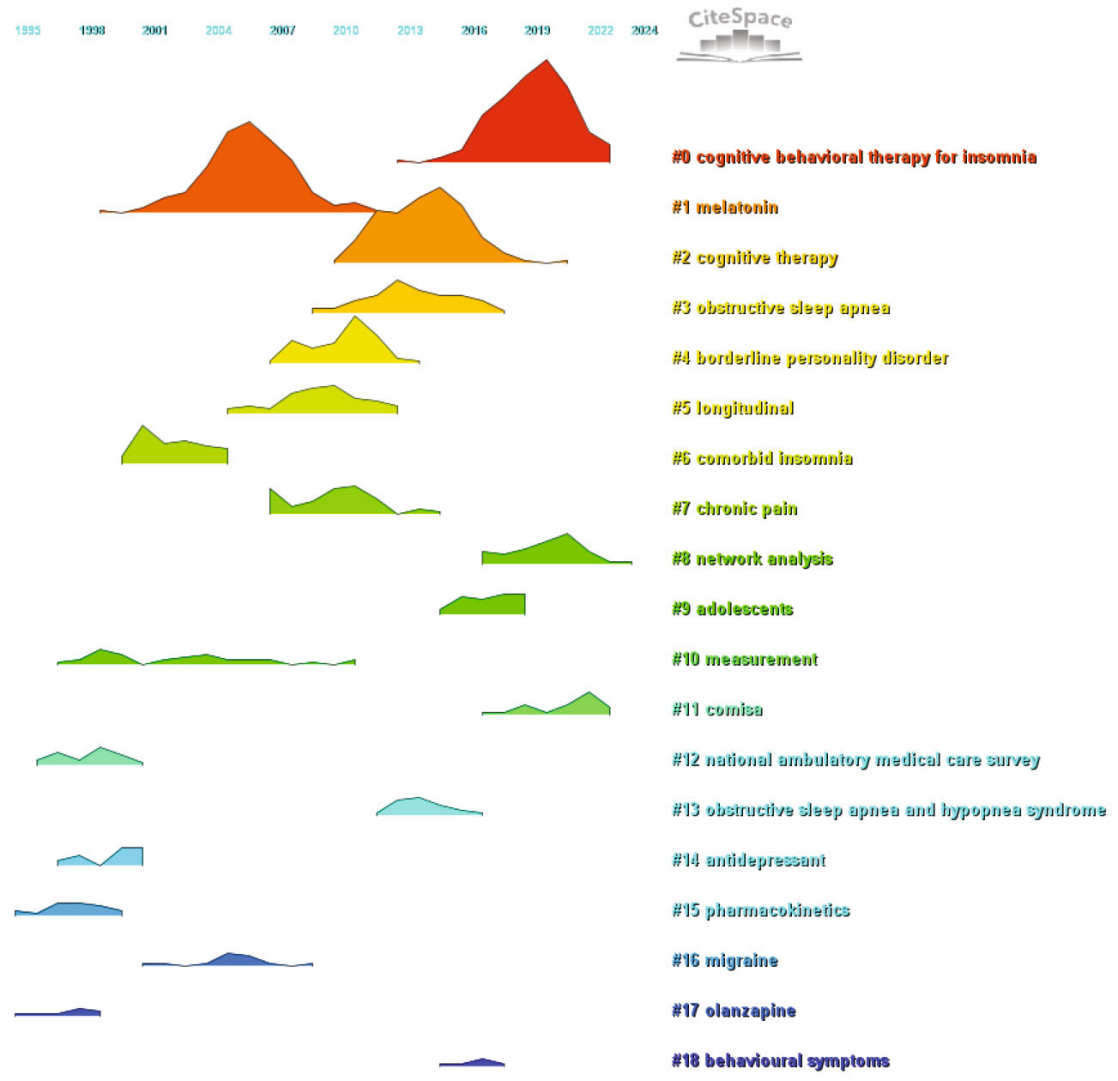
Peak map of cocited reference.

Simultaneously, we conducted cocited reference clustering and temporal clustering analyses (Figures 13 and 14). Our findings indicate that melatonin (Cluster 1), comorbid insomnia (Cluster 6), measurement (Cluster 10), National Ambulatory Medical Survey (Cluster 12), antidepressants (Cluster 13), pharmacokinetics (Cluster 15), migraine (Cluster 16), and olanzapine (Cluster 17) were the focal points of early research in this field. Mid-stage research hotspots included obstructive sleep apnea (Cluster 2), borderline personality disorder (Cluster 4), longitudinal studies (Cluster 5), chronic pain (Cluster 7), adolescents (Cluster 9), obstructive sleep apnea and hypopnea syndrome (Cluster 13), and behavioral symptoms (Cluster 18). Currently, emerging research topics and trends in this field include cognitive behavioral therapy for insomnia (Cluster 0), cognitive therapy (Cluster 2), network analysis (Cluster 8), and COMISA (Cluster 11).

**Figure 15:**
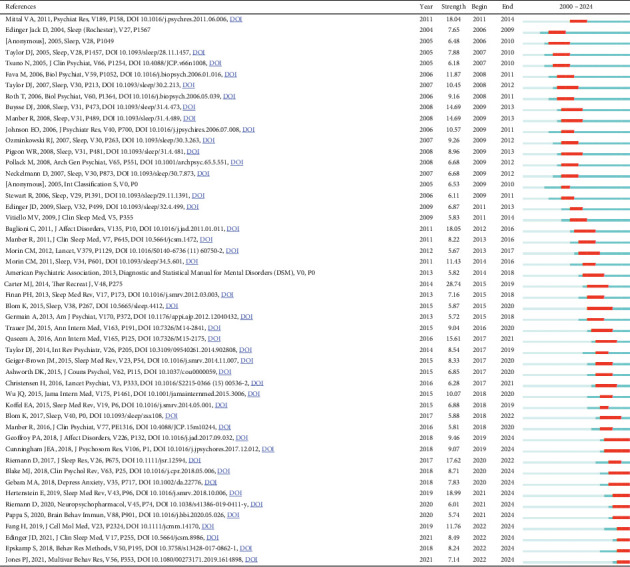
Burst analysis of cited literature.

Finally, we conducted a burst analysis of cocited references using CiteSpace. Burst analysis is a statistical method used to identify references that experience a significant increase in citation frequency over a specific period, highlighting their momentary impact on the field and uncovering emerging concepts and future research trends of interest to scholars. Using this analysis, we identified the 50 most influential citation bursts in the field of insomnia and depression comorbidity. The most highly cited reference was “Insomnia as a Predictor of Mental Disorders: A Systematic Review and Meta-Analysis,” published in *Sleep Medicine Reviews*, which aligns with the results of our previous network analysis of cocited references. All 50 references were published between 2000 and 2024, indicating that these studies have been frequently cited over the past two decades. Notably, there are 11 papers currently at their peak citation period (Figure 15), suggesting that the topic of ICD will continue to attract research interest in the future.

### 3.6. Analysis of Keywords

**Figure 16:**
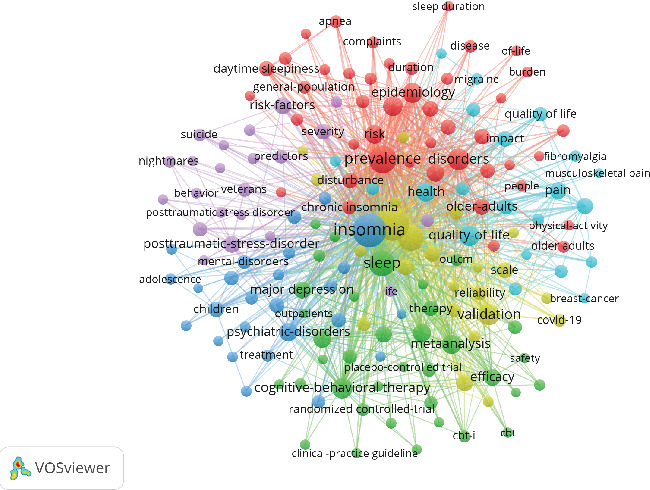
Network map of high-frequency keyword.

**Figure 17:**
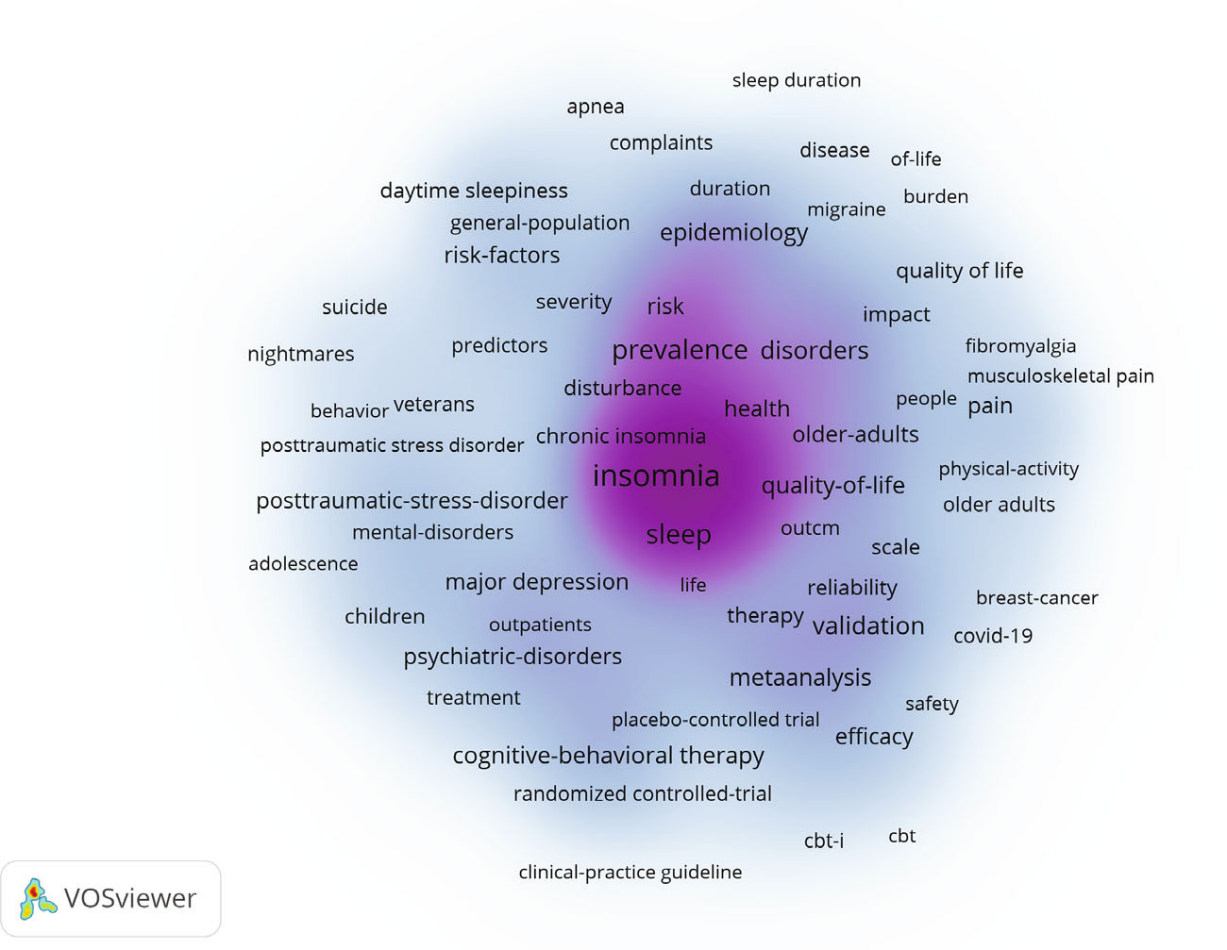
Density map of keyword.

**Figure 18:**
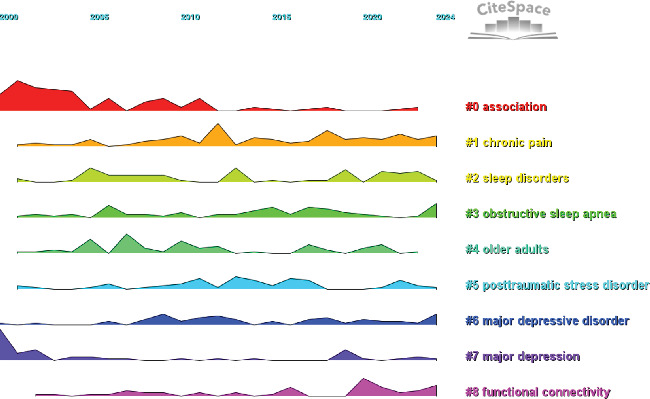
Peak map of keyword.

**Figure 19:**
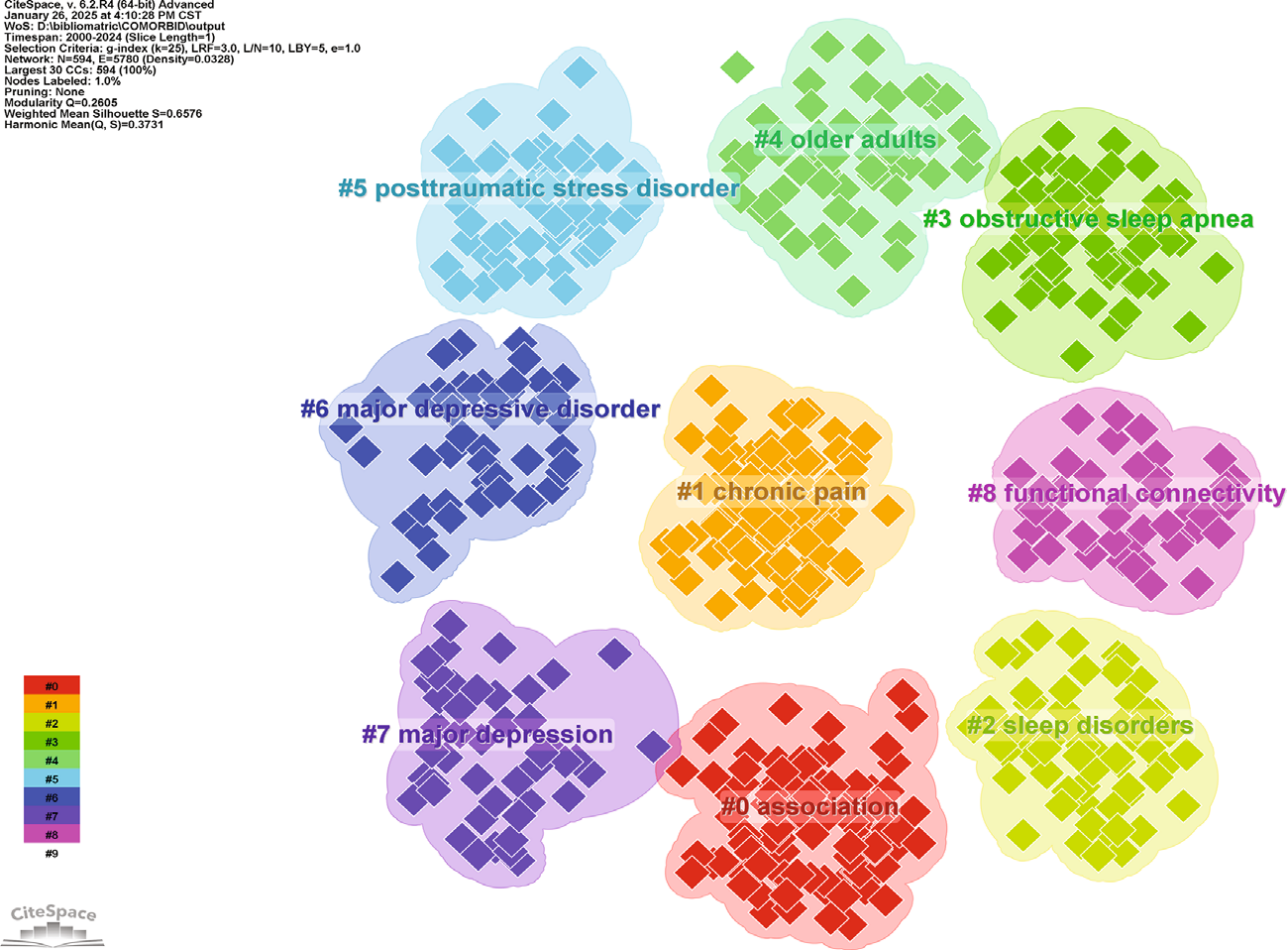
Clustering map of keyword.

**Figure 20:**
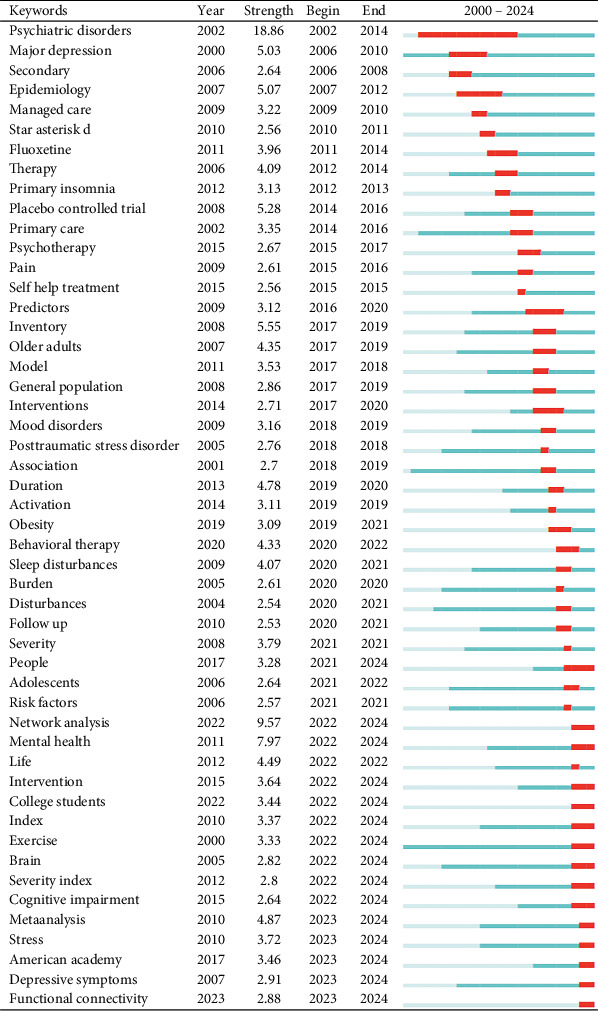
Burst analysis of keywords.

By analyzing keywords, we can quickly gain insights into a research field and its development trends. Based on keyword co-occurrence analysis in VOSviewer, the most frequently occurring keyword is insomnia (*n* = 872), followed by depression (*n* = 854), prevalence (*n* = 464), sleep (*n* = 380), and anxiety (*n* = 362) (Table 8, Figures 16 and 17). After removing irrelevant keywords, we constructed a network comprising 172 keywords that appeared at least 17 times, resulting in six distinct clusters. Cluster 1 (red, 44 keywords) includes prevalence, disorders, association, age, apnea, burden, chronic insomnia, complaint, community, dementia, duration, elderly, impairment, index, obesity, REM sleep, risk factor, population, performance, and sleep quality. Cluster 2 (green, 36 keywords) includes sleep, meta-analysis, CBT, antidepressant, double-blind, fluoxetine, intervention, management, outcome, cognitive behavioral therapy, melatonin, efficacy, safety, trial, and transdiagnostic. Cluster 3 (blue, 26 keywords) includes insomnia, treatment, young adult, substance use, mood, mental disorder, children, DSM-IV, pattern, follow-up, bipolar disorder, and sleep problem. Cluster 4 (yellow, 23 keywords) includes depression, anxiety, actigraphy, reliability, scale, quality index, inventory, instrument, COVID-19, network analysis, questionnaire, sample, stress, validation, version, psychometric property, and inventory. Cluster 5 (purple, 22 keywords) includes alcohol, behavior, ideation, life, mental health, military, nightmares, panic disorder, PTSD, predictors, suicide, veterans, and severity. Cluster 6 (light blue, 21 keywords) includes cancer, diagnosis, disability, distress, fatigue, headache, health, impact, migraine, pain, quality of life, women, and hospital anxiety. Using CiteSpace, we generated peak map plots to visually illustrate the temporal evolution of research hotspots (Figures 18 and 19). Our analysis indicates that association, chronic pain, sleep disorder, obstructive sleep apnea, older adults, posttraumatic stress disorder, major depression, and functional connectivity are the current focal points of research in this field. Additionally, among the 667 strongest burst keywords in this field, we focused on the top 50 keywords with the most significant citation bursts (Figure 20). Notably, emerging research hotspots and potential future directions include network analysis, mental health, intervention, college students, exercise, brain, severity, index, cognitive impairment, meta-analysis, stress, American Academy, depressive symptoms, and functional connectivity. These keywords reflect recent advancements and evolving trends in the study of insomnia and its comorbidities, indicating key areas of interest for future research.

## 4. Discussion

The relationship between insomnia and depression is bidirectional. Study indicates that besides being a common symptom of the disease, insomnia may be one of the significant predictors of the beginning of depression processes [18]. In such a way, people who have insomnia are at substantially greater risk of developing depression. At the same time, inversely, persons experiencing depression are more likely to develop insomnia [20]. Such a comorbid relationship substantially worsens patients' quality of life, complicating both control and treatment of the disease [21]. Therefore, there is a critical need to conduct bibliometric analyses to systematically evaluate research trends and understand the interrelationship between insomnia and depression comorbidity. Such analyses are essential for identifying key research gaps, mapping knowledge evolution, and guiding future clinical interventions.

### 4.1. Performance Analysis: Research Productivity and Trends

Through bibliometric analysis, a three-phase growth pattern in research on ICD from 2000 to 2024 is revealed. We infer that its evolution has been driven by multiple factors, including advancements in academic disciplines, technological progress, and societal demands. During the slow-growth phase (2000–2008), the theoretical framework of ICD remained underdeveloped, as DSM-IV did not establish insomnia as an independent diagnostic category. Moreover, methodological limitations, such as the insufficient spatial resolution of fMRI, hindered the exploration of its pathophysiological mechanisms. In the steady-growth phase (2009–2019), the revision of DSM-5 significantly improved the recognition of ICD [22], and the application of resting-state fMRI deepened the understanding of default mode network dysfunction [23]. The rapid-growth phase (2020 onward) in research on ICD can be attributed to several key factors. Firstly, the recognition of sleep disorders and mental health conditions as critical public health concerns has significantly driven research efforts in this field [24]. The COVID-19 pandemic, which emerged in late 2019, further heightened awareness of these health issues due to the sharp increase in their global burden, thereby accelerating scientific output [25]. Additionally, interdisciplinary research integrating psychiatry, neurology, and sleep medicine has enhanced the understanding of ICD, leading to more comprehensive and nuanced studies [26]. Moreover, increased funding from health organizations and governmental bodies has facilitated large-scale research initiatives, as global health policies increasingly prioritize mental health, recognizing the profound impact of sleep disorders on overall well-being [27]. Furthermore, the advancement of machine learning and multimodal data analysis has enabled more precise ICD subtyping [28], while regulatory progress in digital therapeutics has expanded treatment modalities, further stimulating research activity [29].

From the country and organization co-occurrence, it is evident that the United States leads in research output in this field, highlighting its dominant role in global collaboration. Furthermore, among the top 10 most productive organizations, nine of them are from the United States. University of California System, US Department of Veterans Affairs, and the Veterans Health Administration (VHA) underscore the critical role of government supported medical organizations in advancing research in this domain. We infer that the reason why two of the three most productive and highly cited organizations are related to veterans is that veterans are at high risk for insomnia and depression due to combat exposure, prolonged sleep deprivation, and high-stress conditions. Studies indicate that approximately 40% of veterans experience clinical or subclinical levels of insomnia, which is strongly associated with depression, increased suicide risk, and diminished health functioning [30]. Additionally, the Veterans Administration (VA) possesses the world's largest electronic health database for veterans, providing high-quality data that supports impactful research and further enhances the citation influence of publications in the field of ICD [31]. Lastly, the high policy priority of veterans' mental health issues has led to extensive support from the US government, healthcare organizations, and society, making veteran-related organizations key contributors to research in this area. Despite China ranking second in publication volume, its citation rate per paper remains relatively low, suggesting that Chinese research in this field lacks the global impact compared to the United States.

The five most influential journals in this field are *Sleep Medicine*, *Journal of Affective Disorders*, *Sleep*, *Frontiers in Psychiatry*, and *Journal of Clinical Sleep Medicine*. By summarizing the focus of attention for each journal, we surmised five research trends in the field: advancements in basic research, with increasing focus on the neurobiological mechanisms linking sleep and depression; diversification of clinical interventions, with growing emphasis on nonpharmacological treatments in clinical applications; recognition of insomnia as a critical factor in the course of depression, with more studies identifying it as a core pathophysiological component of depressive disorders; expansion of digital health and emerging technologies, including AI-driven predictive modeling and remote nonpharmacological interventions; and personalized medicine and precision therapy, with research increasingly focusing on individualized interventions and tailored sleep management strategies for specific patient populations. Overall, the research track in this field is shifting towards interdisciplinary integration, personalized treatment approaches, and multimodal interventions, aiming at enhancing both scientific understanding and clinical outcomes.

### 4.2. Science Mapping: Thematic Evolution and Research Hotspots

Science mapping analysis provides insights into the thematic evolution of research on ICD. Based on the results of the dual-map overlay analysis, academic research in this field over the past two decades has involved multiple disciplines, particularly medicine, psychology, health sciences, and social sciences. Journals in the health/nursing/medicine domain have made substantial contributions to ICD field, with the impact of these studies extending to psychology/education//health. Meanwhile, journals in the psychology/education/social domain have had a significant influence on mental health/neuroscience/clinical medicine. Citation path analysis reveals that knowledge flow in the ICD field primarily occurs through three pathways: First, research in the health/nursing/medicine domain is predominantly cited by psychology/education/health, reflecting the significant impact of clinical medicine and health intervention studies on the mental health field. For example, research on pharmacological treatments and nonpharmacological interventions for ICD, such as CBT-i and sleep management strategies, is widely cited in psychology and clinical medicine. Second, research from the psychology/education/social domain is extensively cited by psychology/education/health, neuroscience/sports medicine/ophthalmology, and medicine/clinical medicine, indicating that studies in this area are increasingly extending into neuroscience and clinical applications, especially in functional connectivity and physical interventions. Overall, these citation pathways reveal the interdisciplinary integration and knowledge translation from basic psychology to clinical medicine and neuroscience in the ICD field. This suggests that the field is experiencing a trend of multidisciplinary integration and translational application.

In addition, through analysis to keywords and cocited reference, our findings reveal that early research (2000–2010) predominantly focused on epidemiological associations and psychiatric disorders, as indicated by high-frequency keywords such as “psychiatric disorder,” “prevalence,” and “risk factor.” This suggests that the initial phase of research is aimed at establishing the bidirectional relationship between insomnia and depression, as well as investigating their prevalence and risk factors [32, 33]. From 2011 to 2020, there was a noticeable shift towards clinical management strategies, as evidenced by the increased prominence of keywords such as “CBT-i,” “pharmacological intervention,” and “sleep hygiene.” The growing focus on CBT-i as a primary treatment aligns with a meta-analysis [34], which demonstrated the efficacy of CBT-i in treating both insomnia and depression. Additionally, keyword clustering analysis identified a transition from symptom-based research to treatment-oriented approaches, reflecting a shift in clinical practice. Recently (2020–2024), keyword burst analysis and temporal evolution mapping indicate a growing focus on more diverse and comprehensive intervention strategies, analytical approaches, broader target populations, and neurobiological mechanisms. In summary, the thematic evolution of ICD research shows a progression from epidemiological studies (2000–2010) to treatment-oriented research (2011–2020) and, more recently, to an emphasis on diverse populations, disease mechanisms, and intervention strategies (2020–2024). These findings provide a roadmap for future research, highlighting the need to explore disease mechanisms across different populations and the importance of multimodal interventions.

### 4.3. Network Analysis: Research Collaboration and Knowledge Structure

Our network analysis reveals several important trends in global research collaboration in the field of ICD. The United States continues to dominate the field, forming strong research ties with European countries such as the United Kingdom, Germany, and France. In contrast, China's research network appears more regionally clustered, with stronger collaborations with Japan, South Korea, and Australia, but limited global integration. This finding is consistent with previous studies in one dementia bibliometrics [35], which suggest that Chinese researchers might benefit from expanding their international collaborations to increase their impact.

In terms of author collaboration, network visualization highlights three major, tightly connected collaborative clusters. The green cluster, centered around Allison G. Harvey, has made significant contributions to understanding CBT-i, the bidirectional relationship between insomnia and depression, emotional regulation, and personalized treatment [36]. Their work has not only deepened our understanding of the comorbid relationship between insomnia and depression but also played a crucial role in validating CBT-i as a nonpharmacological intervention for both conditions, making them key figures in developing treatment strategies for this comorbidity. The blue cluster, represented by Charles M. Morin and Susan M. McCurry, is also a proponent of CBT-i, but they advocate for an integrated treatment approach to improve existing therapeutic models [37, 38]. Additionally, they focus on the biological mechanisms of sleep and mood in the context of ICD, contributing to both the foundational research and treatment approaches in the field [39]. The red cluster, centered around Daniel J. Buysse and Jack D. Edinger, has similarly contributed to optimizing CBT-i methods. They advocate that insomnia is not only caused by physiological factors but is also closely related to emotional, cognitive, and behavioral factors [40]. Moreover, they explore the role of neurotransmitters and circadian rhythms in insomnia and depression patients, driving advancements in the understanding and treatment of ICD [41].

### 4.4. Limitations

Our study has several limitations, influenced by certain objective factors. Firstly, the bibliometric analysis software has rigid data requirements, leading to the exclusion of certain article types, such as conference abstracts, news items, and bulletins, which could result in missing some research in the field of ICD. Furthermore, we only screened the publications based on titles, abstracts, and keywords without reviewing the full texts. This approach may cause us to overlook more detailed insights and discussions that could contribute to a deeper understanding of ICD. Finally, while professional software was used to conduct the bibliometric analysis, providing objective quantitative data, the analysis and interpretation of the data inevitably include some degree of subjectivity, and the impact of this subjectivity on the results cannot be entirely eliminated.

## 5. Conclusion

This study using bibliometric analysis on ICD from 2000 to 2024 demonstrates a transition from initial epidemiological studies to research focusing on treatment, particularly regarding neurobiological mechanisms and digital health interventions. The United States leads in global research efforts, with notable contributions also from China and Australia. Renowned researchers like Allison G. Harvey, Charles M. Morin, and Daniel J. Buysse have made significant advancements in this field. However, international collaboration, particularly with China, has the potential to be strengthened. Future research should focus on personalized, multimodal interventions and explore ICD mechanisms across diverse populations, integrating emerging technologies to improve treatment outcomes.

## Figures and Tables

**Table 1:** Summary of the data sources and selection.

**Category**	**Specific standard requirements**
Research database	Web of Science Core Collection
Searching period	January 2000–December 2024
Language	English
Searching keywords	((((((((TS = (Depression)) OR TS = (“Depressive Symptoms”)) OR TS = (“Depressive Symptom”)) OR TS = (“Symptom, Depressive”)) OR TS = (“Emotional Depression”)) OR TS = (“Depression, Emotional”)) OR TS = (“Depressive Disorder”)) OR TS = (“Bipolar Disorder”)) OR TS = (“Dysthymic Disorder”) AND ((((((((((TS = (Insomnia∗)) OR TS = (“Insomnia Disorder”)) OR TS = (“Chronic Insomnia”)) OR TS = (“Nonorganic Insomnia”)) OR TS = (“Insomnia, Nonorganic”)) OR TS = (“Primary Insomnia”)) OR TS = (“Psychophysiological Insomnia”)) OR TS = (“Insomnia, Psychophysiological”)) OR TS = (“Secondary Insomnia”)) OR TS = (“Transient Insomnia”)) OR TS = (“Insomnia, Transient”) AND (TS = (“comorbid”) OR TS = (“comorbidity”) OR TS = (“co-occurrence”))
Data extraction	Export with full records and cited references in plain text format
Sample size	1767

**Table 2:** Country published literature.

**Rank**	**Country/region**	**Article counts**	**Centrality**	**Percentage (%)**	**Citation**	**Citation per publication**
1	United States	709	0.33	43.66%	32894	46.39
2	China	269	0.15	16.56%	5224	19.42
3	Australia	111	0.03	6.83%	4560	41.08
4	United Kingdom	110	0.13	6.77%	4024	36.58
5	Canada	106	0.06	6.53%	7007	66.10
6	Sweden	75	0.04	4.62%	4948	65.97
7	Italy	65	0.1	4.00%	4634	71.29
8	Japan	63	0.04	3.88%	1269	20.14
9	South Korea	62	0.08	3.82%	1429	23.05
10	Germany	61	0.13	3.76%	5406	88.62

**Table 3:** Organization published literature in the ICD.

**Rank**	**Organization**	**Country**	**Number of studies**	**Total citations**	**Average citation**
1	University of California System	United States	93	4045	43.49
2	US Department of Veterans Affairs	United States	92	4240	46.09
3	Veterans Health Administration (VHA)	United States	86	3048	35.44
4	Harvard University	United States	70	2911	41.59
5	Pennsylvania Commonwealth System of Higher Education (PCSHE)	United States	68	3525	51.84
6	University of Pittsburgh	United States	50	1659	33.18
7	University of London	England	46	1345	29.24
8	Harvard Medical School	United States	39	1551	39.77
9	Stanford University	United States	37	2657	71.81
10	Karolinska Institutet	United States	36	699	19.42

**Table 4:** Top 10 journals by publication volume on ICD.

**Rank**	**Journal**	**Article counts**	**Percentage (1624)**	**IF**	**Quartile in category**
1	*Sleep Medicine*	74	4.56%	3.8	Q1
2	*Journal of Affective Disorders*	56	3.45%	4.9	Q1
3	*Sleep*	52	3.20%	5.3	Q1
4	*Frontiers in Psychiatry*	36	2.22%	3.2	Q2
5	*Journal of Clinical Sleep Medicine*	31	1.91%	3.5	Q1
6	*Behavioral Sleep Medicine*	27	1.66%	2.2	Q3
7	*Journal of Psychiatric Research*	26	1.60%	3.7	Q1
8	*Journal of Sleep Research*	24	1.48%	3.4	Q2
9	*Psychiatry Research*	24	1.48%	4.2	Q1
10	*Sleep Medicine Reviews*	22	1.35%	11.2	Q1

**Table 5:** Top 10 journals by citation count for articles on ICD.

**Rank**	**Cited journal**	**Cocitation**	**IF (2023)**	**Quartile in category**
1	*Sleep*	1149	5.3	Q1
2	*Sleep Med*	911	3.8	Q1
3	*Sleep Med Rev*	856	11.2	Q1
4	*J Affect Disorders*	761	4.9	Q1
5	*Am J Psychiat*	663	15.1	Q1
6	*J Clin PsychiaT*	656	4.5	Q1
7	*Psychiat Res*	652	4.2	Q1
8	*J Psychosom Res*	622	3.5	Q2
9	*JAMA-J Am Med Assoc*	582	63.5	Q1
10	*Arch Gen Psychiat*	578	—	—

**Table 6:** Author's publications and cocitation.

**Rank**	**Author**	**Count**	**Rank**	**Cocited author**	**Citation**
1	Allison G. Harvey	24	1	Daniel J. Buysse	499
2	Charles M. Morin	15	2	Charles M. Morin	485
3	Thomas Roth	13	3	Maurice M. Ohayon	416
4	Vincent Mysliwiec	11	4	Celyne H. Bastien	324
5	Daniel J. Buysse	10	5	Ronald C. Kessler	285
6	Christopher L. Drake	10	6	Dieter Riemann	250
7	Susanna Jernelöv	10	7	Daniel J. Taylor	247
8	Daniel J. Taylor	10	8	Kurt Kroenke	214
9	Jack D. Edinger	9	9	Allison G. Harvey	212
10	Rachel Manber	9	10	Chiara Baglioni	197

**Table 7:** Top 10 cocited reference in the ICD from 2000 to 2024.

**Rank**	**Title**	**Journal**	**Author(s)**	**Total citations**
1	Insomnia as a Predictor of Mental Disorders: A Systematic Review and Meta-Analysis	Sleep Med Rev	Hertenstein E.	50
2	Insomnia as a Predictor of Depression: A Meta-Analytic Evaluation of Longitudinal Epidemiological Studies	J Affect Disorders	Chiara Baglioni	38
3	Management of Chronic Insomnia Disorder in Adults: A Clinical Practice Guideline From the American College of Physicians	Ann Intern Med	Amir Qaseem	37
4	Depression in Sleep Disturbance: A Review on a Bidirectional Relationship, Mechanisms and Treatment	J Cell Mol Med	Hong Fang	34
5	European Guideline for the Diagnosis and Treatment of Insomnia	J Sleep Res	Dieter Riemann	34
6	Cognitive Behavioral Therapy for Insomnia Enhances Depression Outcome in Patients With Comorbid Major Depressive Disorder and Insomnia	Sleep	Rachel Manber	31
7	Prevalence, Course, and Comorbidity of Insomnia and Depression in Young Adults	Sleep	Daniel J. Buysse	31
8	Insomnia and Hypersomnia in Major Depressive Episode: Prevalence, Sociodemographic Characteristics and Psychiatric Comorbidity in a Population-Based Study	J Affect Disorders	Pierre A. Geoffroy	24
9	Cognitive Behavioural Therapy for Insomnia (CBT-i) to Treat Depression: A Systematic Review	J Psychosom Res	Jasmyn E. A. Cunningham	23
10	The Insomnia Severity Index: Psychometric Indicators to Detect Insomnia Cases and Evaluate Treatment Response	Sleep	Charles M. Morin	23

**Table 8:** Top 10 high frequency keywords.

**Rank**	**Keyword**	**Counts**
1	Insomnia	872
2	Depression	854
3	Prevalence	464
4	Sleep	380
5	Anxiety	362
6	Validation	193
7	Cognitive-behavioral therapy	174
8	Quality-of-life	173
9	Epidemiology	156
10	Association	146
11	Metaanalysis	141
12	Risk	131
13	Psychiatric-disorders	130
14	Health	127
15	Sleep disturbance	115
16	Major depressive disorder	110
17	Adults	104
18	Efficacy	98
19	Pain	98
20	Major depression	96

## Data Availability

The data that support the findings of this study are available from the corresponding authors upon reasonable request.
